# Resveratrol and acetyl-resveratrol modulate activity of VEGF and IL-8 in ovarian cancer cell aggregates via attenuation of the NF-κB protein

**DOI:** 10.1186/s13048-016-0293-0

**Published:** 2016-12-01

**Authors:** Alexandria B. Tino, Kenny Chitcholtan, Peter H. Sykes, Ashley Garrill

**Affiliations:** 1 Department of Obstetrics and Gynaecology, University of Otago, Christchurch, 2 Riccarton Avenue, Christchurch, 8011 New Zealand; 2 Obstetrics and Gynaecology Department Christchurch Women’s Hospital, Private Bag 4711, Christchurch, 8140 New Zealand; 3 School of Biological Sciences, University of Canterbury, Private Bag 4800, Christchurch, 8140 New Zealand

**Keywords:** Ovarian cancer, Resveratrol, Acetyl-resveratrol, VEGF, NF-κB, Cell clusters, IL-8

## Abstract

**Background:**

Key features of advanced ovarian cancer include metastasis via cell clusters in the abdominal cavity and increased chemoresistance. Resveratrol and derivatives of resveratrol have been shown to have antitumour properties. The purpose of this study was to investigate the effect of resveratrol and acetyl-resveratrol on 3D cell aggregates of ovarian cancer, and establish if NF-κB signalling may be a potential target.

**Methods:**

Poly-HEMA coated wells were used to produce 3D aggregates of two ovarian cancer cell lines, SKOV-3 and OVCAR-5. The aggregates were exposed to 10, 20 or 30 μM resveratrol or acetyl-resveratrol for 2, 4 or 6 days. Cell growth and metabolism were measured then ELISA, western blot and immunofluorescence were utilised to evaluate VEGF, IL-8 and NF-κB levels.

**Results:**

Resveratrol and acetyl-resveratrol reduced cell growth and metabolism of SKOV-3 aggregates in a dose- and time-dependent manner. After 6 days all three doses of both compounds inhibited cell growth. This growth inhibition correlated with the attenuated secretion of VEGF and a decrease of NF-κB protein levels. Conversely, the secretion of IL-8 increased with treatment. The effects of the compounds were limited in OVCAR-5 cell clusters.

**Conclusions:**

The results suggest that resveratrol and its derivative acetyl-resveratrol may inhibit in vitro 3D cell growth of certain subtypes of ovarian cancer, and growth restriction may be associated with the secretion of VEGF under the control of the NF-κB protein.

## Background

Ovarian cancer is a lethal gynaecological cancer and is the seventh most common cause of cancer death among women [1]. The majority of women present with an advanced stage of the disease [2]. The current treatment options of debulking surgery and chemotherapy are generally not curative in advanced stages of the disease due to recurrence and chemoresistance [3]. Therefore, alternative treatments that target cancer cells, reduce tumour growth and increase tumour-free survival are of great importance.

Ovarian cancer metastasises via the fluid in the peritoneal cavity. Cells slough off the primary tumour and form small 3D clusters or aggregates in the peritoneal fluid. The accumulation of peritoneal fluid, which is known as ascites, is often associated with advanced ovarian cancer and correlates with poor prognosis [4]. The microenvironment of the ascitic fluid is rich in a wide range of growth factors and cytokines, and these are believed to sustain cell cluster survival, growth and secondary site establishment [5]. Relatively little is known, however, about the interactions between ascitic fluid components and the 3D aggregates. The 3D aggregates of ovarian cancer cells are integral to metastasis, and are possibly involved with the development of chemoresistance [6]. Few studies have investigated the use of potential therapeutic agents against the 3D aggregates of ovarian cancer.

Our knowledge of ovarian cancer aggregate survival in ascitic fluid is limited. However, studies on other types of solid tumour, coupled with analyses of pertinent proteins suggest that angiogenic and inflammatory mediators may play a significant role. Of the numerous pro-angiogenic cytokines vascular endothelial growth factor (VEGF) is one of the most well described. In addition to being a key regulator of angiogenesis, it also enhances cell survival, proliferation and migration [7, 8]. Studies have revealed that VEGF is over expressed by ovarian cancer [9, 10]. Interleukin-8 (IL-8) is another regulation protein involved in tumorigenic activities in cancers, and has been reported to be over expressed in ovarian cancer [11–13], suggesting its importance to ovarian cancer carcinogenesis.

There is evidence that VEGF and IL-8 expression in ovarian cancer are under the transcriptional control of nuclear factor kappaB (NF-κB) [14]. The NF-κB family of transcription factors are activated via two signalling pathways [15]. In normal cells, NF-κB activation is very tightly regulated, but constitutive activation has been identified in a range of cancers [16–18] suggesting that NF-κB signalling may be important in cancer survival. Furthermore, in some cancer types the activation of NF-κB correlates with the expression of VEGF [19] and IL8 [20]. However, this correlation is not well understood in ovarian cancer.

The polyphenol resveratrol is a possible inhibitor of the NF-κB signalling pathway in ovarian cancer. Resveratrol is one of the major antioxidants found in the skin of red grapes and has anti-inflammatory [21], cardioprotective [22] and anti-carcinogenic properties [23]. It has been linked to the inhibition of NF-κB in prostate [24] and lung cancer [25], and the down regulation of VEGF [26] and IL-8 [27]. However, there have been no reports on the effects of resveratrol on NF-κB activity, cytokine expression or their correlation with the growth of ovarian cancer clusters.

Although resveratrol appears to be a very promising cancer treatment, it has low bioavailability [28, 29], because of this the resveratrol derivative acetyl-resveratrol has aroused interest. In chemopreventive and chemotherapeutic studies, it appears to possess the same characteristics as resveratrol, but may not undergo such rapid metabolism in the liver and with increased cellular uptake may have greater bioavailability [30]. The hydroxyl groups of resveratrol are acetylated in acetyl-resveratrol which accounts for it being a more stable compound and increased uptake in the body [31].

In the present study, we examined the effect of resveratrol and acetyl-resveratrol on cell growth and on the production of regulatory factors in 3D aggregates of two ovarian cancer cell lines. Our data shows that both compounds significantly inhibit cell growth, VEGF secretion and NF-κB activation in a time-, dose- and cell line dependent manner. The secretion of IL-8 increased.

## Methods

### Cell culture

The human ovarian adenocarcinoma cell lines SKOV-3 and OVCAR-5 were obtained from Dr. Judith McKenzie, Haematology Research group, University of Otago, Christchurch, New Zealand. Both cell lines were maintained in Dulbecco’s Modified Eagle Medium (GIBCO®, Life Technologies, New Zealand), supplemented with 10% fetal bovine serum (GIBCO®, Life Technologies, New Zealand), PenStrep (GIBCO®, Life Technologies, New Zealand) at a working concentration of 100 units/ml penicillin and 100 μg/ml streptomycin, 2 mM GlutaMAX™ (GIBCO®, Life Technologies, New Zealand) and 2 μg/ml Fungizone® (Life Technologies, New Zealand). The supplemented media is henceforth referred to as working media. SKOV-3 and OVCAR-5 cells in working media were continuously maintained in a culture flask at 37 °C in a humidified 5% CO_2_ atmosphere incubator.

### Production of 3D aggregates

To prevent adhesion of cells to culture plates, 12-well culture plates were pre-coated with 24 mg/ml Polyhydroxyethylmethacrylate (poly-HEMA) (Sigma, New Zealand) prior to cell culturing (0.5 ml/well). Prior to coating, poly-HEMA was fully dissolved in 95% ethanol at a concentration of 24 mg/ml and was heated to approximately 70 °C. After the poly-HEMA was placed in the wells the plates were left overnight at 37 °C on an orbital shaker. Prior to cell culture, the coated wells were washed once with PBS at pH 7.4. To detach the cell monolayer of the SKOV-3 and OVCAR-5 cell lines from the flask surface, they were incubated with 1x trypsin-EDTA for 20–30 min. Cells were counted with a haemocytometer to determine the concentration of the cells in suspension. Cells were then plated at a density of 200,000 cells/well and were incubated at 37 °C in a humidified 5% CO_2_ atmosphere for 6 days. Over this time the cells became clusters and aggregates. Working media was refreshed every 2 days with 1 ml of fresh working media.

### Treatment with resveratrol, acetyl-resveratrol and Bay 11-7085

Resveratrol and acetyl-resveratrol were provided by Dr. Saurabh Shah, Biotivia (USA). A NF-κB inhibitor, Bay 11-7085, was purchased Sigma-Aldrich (Auckland, New Zealand). Resveratrol was dissolved in a 50:50 combination of PBS and DMSO, acetyl-resveratrol and Bay 11-7085 were dissolved in 100% DMSO. Fresh working media containing the relevant compounds (at concentrations of 10, 20 and 30 μM) was replaced every 2 days for up to 6 days. Thus, at the endpoint of culturing the cells had a total incubation time of 8, 10 or 12 days respectively; 6 days developing the aggregates and 2, 4 or 6 days of treatment. For all experiments, the final concentration of DMSO used in controls was the concentration of DMSO that was present in the 30 μM treatments. At least 4 independent experiments were carried out for each treatment and within each experiment there were 3 replicates.

### Growth determination using the crystal violet assay

Growth of the 3D aggregates was quantified indirectly using crystal violet staining. In brief, cell aggregates were isolated and incubated with 1x trypsin-EDTA for 20 min at 37 °C. Cells were washed twice with PBS (pH 7.4) and were incubated for a further 15 min at 37 °C with 2 mg/ml crystal violet in 2% (v/v) ethanol in milliQ water. The cells were then washed with milliQ water to remove unbound crystal violet, and were isolated by centrifugation at 1500 rpm for 5 min. The supernatant was removed and the process repeated 3 times until the supernatant was colourless. Cells were then lysed in 10% (w/v) sodium dodecyl sulphate (SDS) solution. 200μl of the homogenous cell lysate was then loaded onto a 96-well plate. The optical density was determined at 560 nm (OD_560_) using a microplate reader (SpectraMax M5, Molecular Devices).

### Cellular metabolism determination using the Alamar blue assay

On the 6^th^ day of treatment with compounds, 0.5 ml media was removed from each well and 50 μl of Alamar blue dye (ThermoFisher Scientific, New Zealand) added to cell aggregates. Aggregates were incubated at 37 °C with the dye for 4 h after which 200 μl of media from each well was transferred to a 96-well plate. The absorbance at 570 and 600 nm was measured using a microplate reader (SpectraMax M5, Molecular Devices). Cellular metabolism was calculated from the difference of absorbance at 600 and 570 nm.

### Detection of vascular endothelial growth factor (VEGF) and interleukin-8 (IL-8)

The conditioned media of the control and treated cells (2, 4 or 6 days) was used to determine secreted VEGF and IL-8 levels. The conditioned media were centrifuged at 1500 rpm to remove cell debris and stored at -80 °C until assayed using the DuoSet Human VEGF ELISA kit (R&D System, New Zealand) and the DuoSet Human IL-8 ELISA kit (R&D System, New Zealand). The assays were carried out according to the manufacturer’s instructions. Samples were diluted 50%, the amount of VEGF or IL-8 was determined by comparing absorbance of each well to a standard curve and corrected for total protein.

### Western blotting

Cell aggregates were harvested after their respective treatments by centrifugation at 1500 rpm for 5 min and the cell pellet was resuspended in cold RIPA buffer containing protease inhibitor cocktail tablets (Complete Mini, Roche, New Zealand). The cell lysates were left on ice for 30 min to ensure total cell lysis. Total protein was determined using a Pierce™ BCA protein assay kit (ThermoFisher Scientific, New Zealand) according to the manufacturer’s instructions. Sample buffer (0.2% (v/v) bromophenol blue, 25% (v/v) glycerol, 10% SDS in Tris-HCl and pH 6.8) was added and protein lysates were boiled for 10 min. Samples (10 μg protein) were fractionated in 5–12% SDS-PAGE gels and transferred to PDVF membranes (Bio-Rad Laboratories, Hercules, USA). The markers used were MagicMark Western Protein Standard (Thermofisher, New Zealand) and Precision Plus Protein Standard (Bio-Rad, Hercules, New Zealand). The membranes were blocked for 60 min with either 5% (w/v) nonfat skim milk (Pams brand, New World, New Zealand) or 1% (w/v) bovine serum albumin (ThermoFisher Scientific, New Zealand) in 1% tween-TBS. Antibodies were diluted to 1/500 to 1/1000 with the appropriate blocking solution, and membranes were incubated with primary antibodies overnight at 4 °C. Membranes were developed using either donkey anti-mouse IgG-AP or goat anti-rabbit IgG-AP secondary antibodies (Santa Cruz, CA, USA, 1:10,000). Antibody localisation was determined using a chemiluminescent detection kit (Amersham ECL Prime Western Blotting Detection Reagent Kit, GE Healthcare) and the bands visualized using Alliance 4.7, Unitec (Cambridge, UK). The primary antibodies used were PCNA (sc-25280), pIκBα (sc-8404), NF-κB (sc-372), pNF-κB (sc-33020) and GAPDH (sc-25778), all purchased from Santa Cruz Biotechnology Inc. (Santa Cruz, CA, USA).

### Immunofluorescence

Cell aggregates were harvested after treatments, they were then centrifuged and the supernatant removed. 1 ml of ice cold mixture of methanol:acetone (50:50% (vol/vol)) solution was added to the OVCAR-5 and SKOV-3 cell aggregates and the samples were stored at −20 °C until analysis. Prior to sectioning, SKOV-3 aggregates were stained with the aniline blue dye solution (1% in water (Sigma-Aldrich LTD, New Zealand)) for 20 min and washed with PBS twice. The stained SKOV-3 aggregates were then imbedded in CryO-Z-T solution (Ted Pella Inc., USA) and frozen overnight at -80 °C. SKOV-3 samples were then sectioned into 7 μM thick slices, two slices were placed on appropriately labelled Superfrost plus slides (Menzel-glaser, Germany), 6 slices per sample were collected, and stored at -20 °C until analysis.

The OVCAR-5 cell clusters were removed from the methanol/acetone solution, washed with PBS pH 7.4 and placed onto slides. Slides of both cell lines were dried and blocking buffer added for 1 h at room temperature. Primary antibodies to detect NF-κB or pNF-κB proteins were diluted to 1:200 in 2% BSA in PBS, added to the slides and the slides were incubated overnight at 4 °C. The secondary antibody goat anti-rabbit IgG conjugated with Atto 594 nm (Sigma-Aldrich LTD, New Zealand, 1:1000) was added to slides which were then incubated for 1 h at 37 °C, and then samples were stained with 10 μg/ml Hoechst (Invitrogen, New Zealand) for 20 min. The slides were washed extensively with PBS + 0.1% Tween-20, pH 7.4. Cells were observed and imaged with an epifluorescence microscope (AxioVision 4.5. Apotome software, Carl Zeiss, Oberkochen, Germany).

### Statistical analysis

At least four independent experiments were carried out for each treatment and these were statistically analyzed using GraphPad Prism software (La Jolla, CA, USA). *p* < 0.05 was considered to indicate statistical significance determined by one way ANOVA. All data are presented as mean ± SEM.

## Results

### Attenuation of cellular growth and metabolism by resveratrol and acetyl-resveratrol

**Fig. 1:**
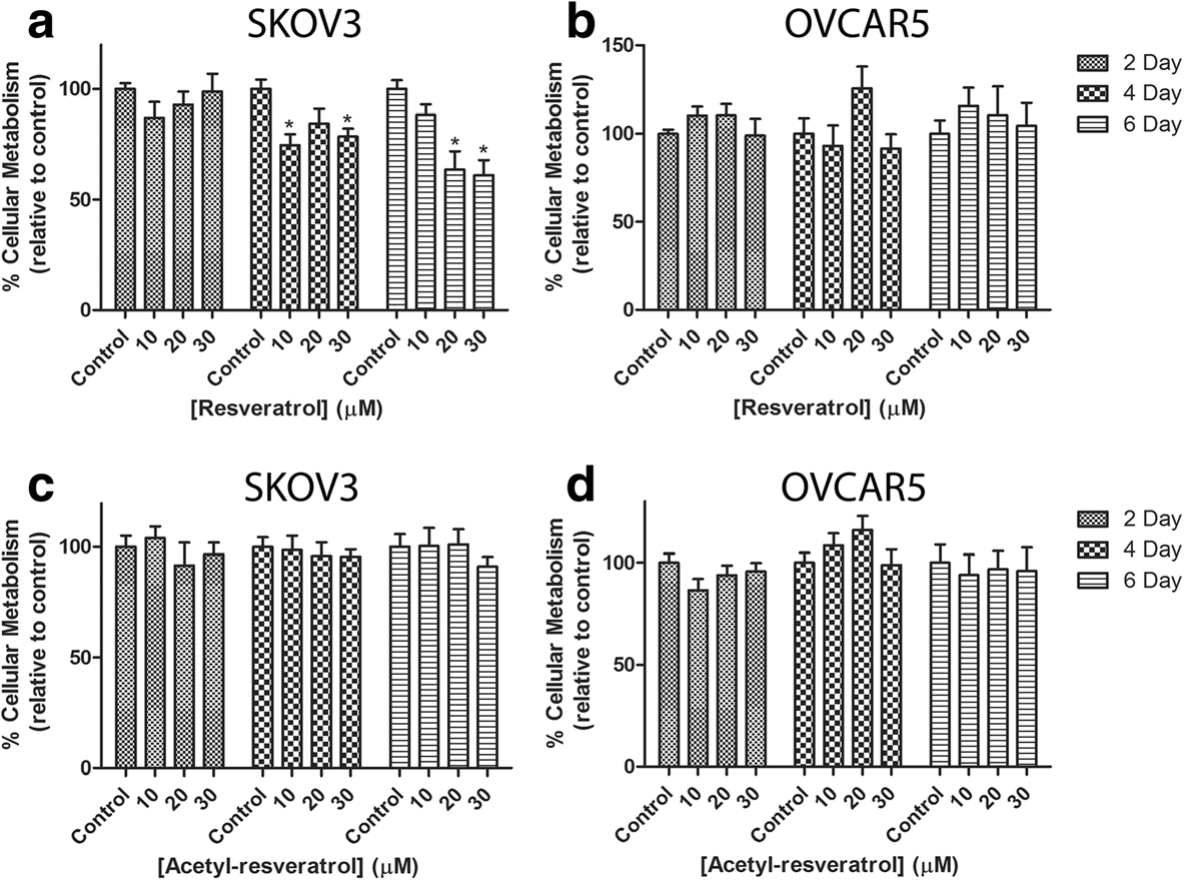
Cellular metabolism relative to control of SKOV-3 aggregates after treatment with resveratrol (**a**) or acetyl-resveratrol (**c**) for 2, 4 or 6 days. Cellular metabolism relative to control of OVCAR-5 clusters after treatment with resveratrol (**b**) or acetyl-resveratrol (**d**) for 2, 4 or 6 days. * indicates statistical significance *p* < 0.05 compared to the control. *n =* 4 independent experiments in triplicate

It has been previously shown that resveratrol reduces the growth of monolayer and clusters of ovarian cancer cell lines [32]. We further examined this in 3D cell aggregates using concentrations of resveratrol and acetyl-resveratrol that were closer to levels found in human serum [29], using the Alamar blue dye assay (Fig. 1). The metabolism of the SKOV-3 aggregates was significantly reduced by between 20 and 40% after 4 and 6 days exposure to resveratrol (Fig. 1a). In contrast, after 2, 4 and 6 days of treatment the OVCAR-5 clusters were unaffected (Fig. 1b). Likewise, acetyl-resveratrol did not have an effect on either cell line (Fig. 1c and d).

**Fig. 2:**
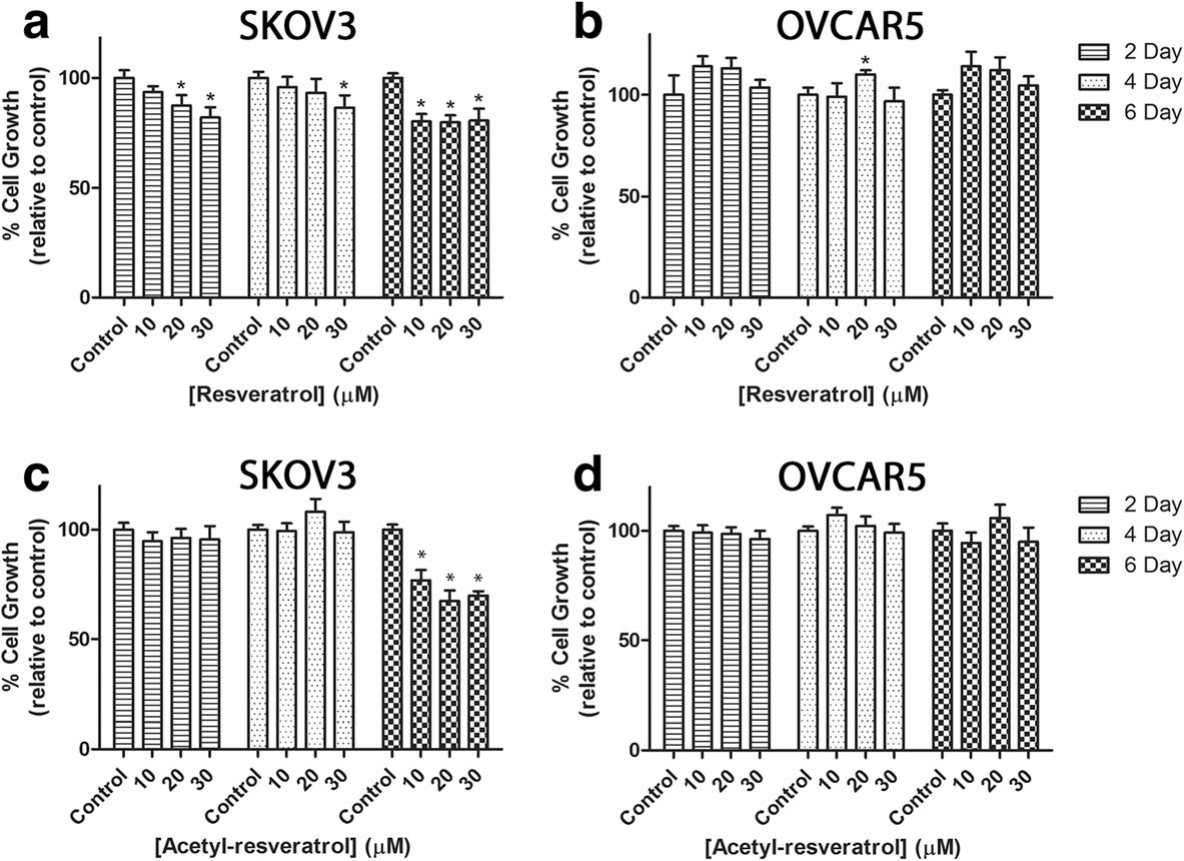
Cell growth relative to control of SKOV-3 aggregates after treatment with resveratrol (**a**) or acetyl-resveratrol (**c**) for 2, 4 or 6 days. Cell growth relative to control of OVCAR-5 clusters after treatment with resveratrol (**b**) or acetyl-resveratrol (**d**) for 2, 4 or 6 days. * indicates statistical significance *p* < 0.05 compared to the control. *n* = 4 independent experiments in triplicate

Next, we investigated the effect of the compounds on cell growth (Fig. 2) by using the crystal violet assay. SKOV-3 cell growth in 3D cell aggregates was significantly decreased after 2 and 4 days of treatment with 30 μM resveratrol and after 6 days treatment at all three concentrations tested (Fig. 2a). It had no effect on growth of OVCAR-5 cell clusters (Fig. 2b). Similarly, acetyl-resveratrol significantly decreased the growth of SKOV-3 cells after 6 days treatment at each of the concentrations tested but had no effect on the OVCAR-5 cells (Fig. 2c and d). Both resveratrol and acetyl-resveratrol did not increase apoptotic cells in both cell lines (data not shown).

### Attenuation of Vascular Endothelial Growth Factor (VEGF) production by resveratrol and acetyl-resveratrol

**Fig. 3:**
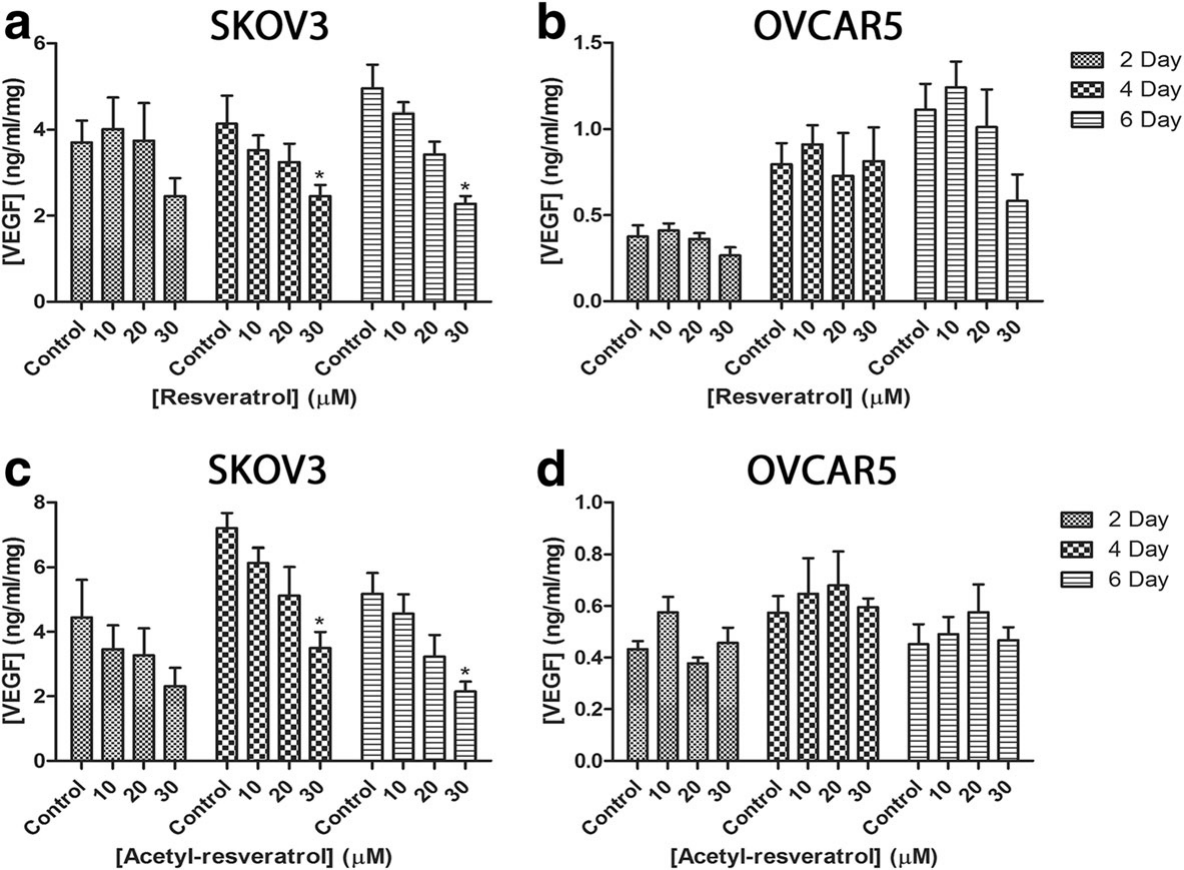
The secretion of vascular endothelial growth factor (VEGF) of SKOV-3 aggregates after treatment with resveratrol (**a**) or acetyl-resveratrol (**c**) for 2, 4 or 6 days. The secretion of VEGF of OVCAR-5 clusters after treatment with resveratrol (**b**) or acetyl-resveratrol (**d**) for 2, 4 or 6 days. * indicates statistical significance *p* < 0.05 compared to the control. *n* = 4 independent experiments in triplicate

VEGF is a glycoprotein that is a key regulator of angiogenesis. Angiogenesis is fundamental to the development of solid cancerous tumours as oxygen and nutrients are required for the cancer to flourish [33]. Elevated levels of VEGF have been observed in ascitic fluid and these correlate with a poor prognosis [34]. To assess the effect of the resveratrol and acetyl-resveratrol on the production of VEGF we used an ELISA. Resveratrol at a concentration of 30 μM reduced VEGF production in SKOV-3 cell aggregates by 41 and 54% after 4 and 6 days respectively (Fig. 3a). Similar results were observed with acetyl-resveratrol (reductions of 49 and 58% respectively). The basal secretion of VEGF by OVCAR-5 clusters was much lower than for SKOV-3 cells, between 0.4 and 1.1 ng/ml/mg (Fig. 3b and d) and the secretion levels were unchanged in the presence of both resveratrol (Fig. 3b) and acetyl-resveratrol (Fig. 3d).

### Resveratrol and Acetyl-resveratrol increase the secretion of Interleukin-8 (IL-8)

**Fig. 4:**
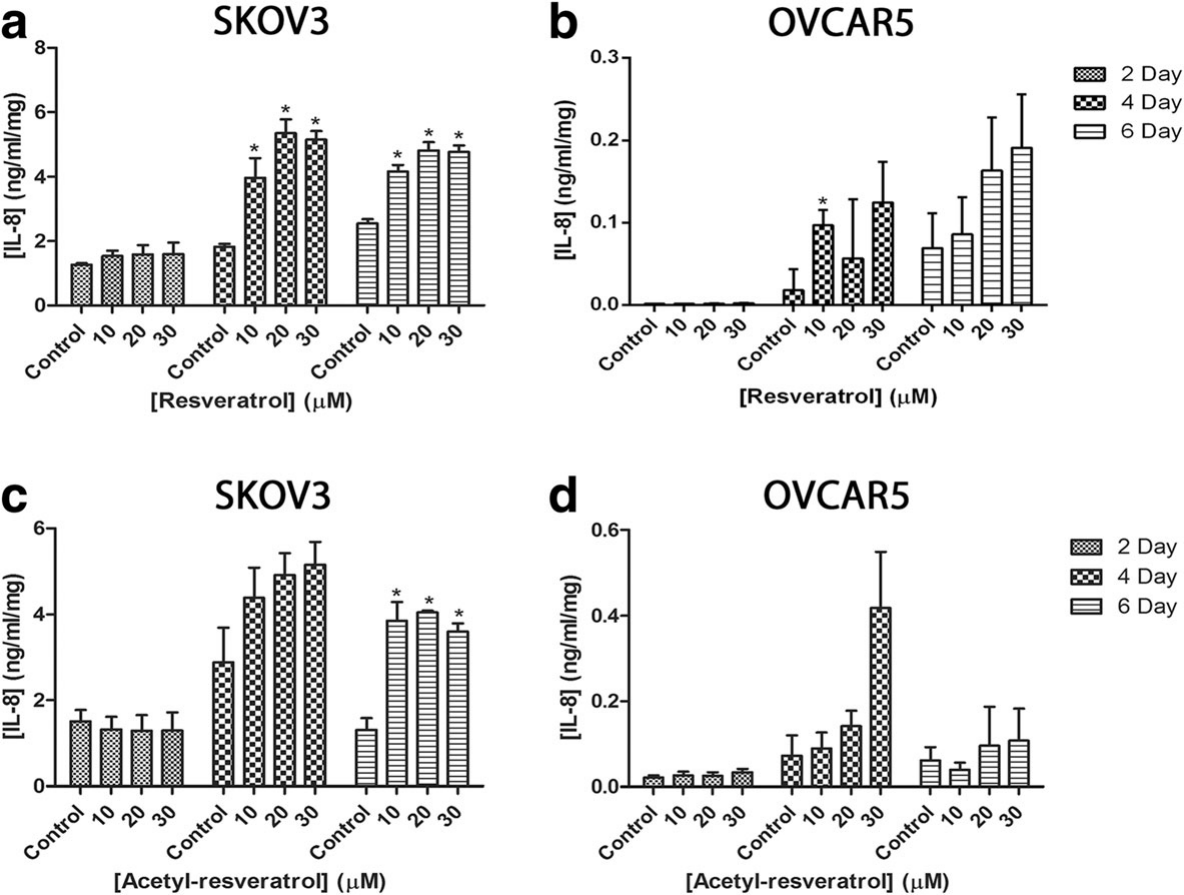
The secretion of interleukin-8 (IL-8) of SKOV-3 aggregates after treatment with resveratrol (**a**) or acetyl-resveratrol (**c**) for 2, 4 or 6 days. The secretion of IL-8 of OVCAR-5 clusters after treatment with resveratrol (**b**) or acetyl-resveratrol (**d**) for 2, 4 or 6 days. * indicates statistical significance *p* < 0.05 compared to the control. *n* = 4 independent experiments in triplicate

The chemokine IL-8 is induced by inflammatory stimuli and is responsible for activation of neutrophils under normal conditions [35]. There is evidence of IL-8 sustaining cancer cell survival [36] and it is over expressed in ovarian cancer [13, 37]. We used an ELISA to gauge the effects of resveratrol and acetyl-resveratrol and found that the compounds caused an increase in the production of IL-8 in both cell lines (Fig. 4). Resveratrol more than doubled the level of IL-8 produced by SKOV-3’s cell aggregates at all three concentrations after 4 and 6 days (Fig. 4a) and an increasing trend was also observed in OVCAR-5 clusters (Fig. 4b). Acetyl-resveratrol also had a profound effect on SKOV-3 IL-8 secretion (Fig. 4c) and again an increasing trend was observed in OVCAR-5 (Fig. 4d).

### Effects of resveratrol and acetyl-resveratrol on the expression and activation of NF-κB protein

**Fig. 5:**
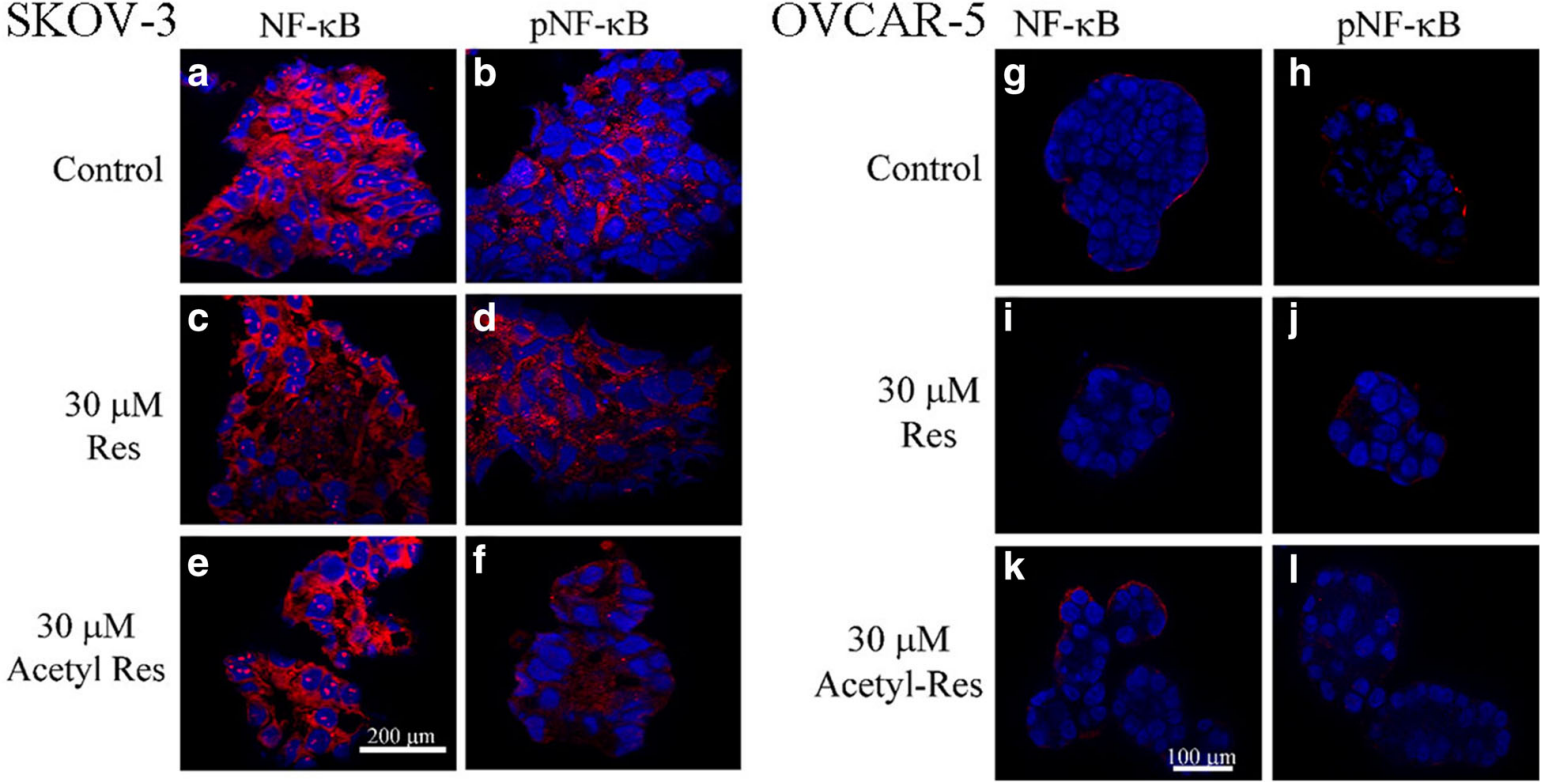
Immunofluorescent images of NF-κB and pNF-κB in frozen cut sections of SKOV-3 control aggregates (**a** and **b**) treated with resveratrol (**c** and **d**) or acetyl-resveratrol (**e** and **f**) for 6 days. NF-κB and pNF-κB in OVCAR-5 control clusters (**g** and **h**) treated with resveratrol (**i** and **j**) or acetyl-resveratrol (**k** and **l**) for 6 days. NF-κB and pNF-κB were stained red and nuclei were stained blue

The signalling pathways that control cancer cell survival and proliferation are of great interest for targeted cancer cell therapy. There are many cascades involved in cancer cell growth, but a prominent one, the Nuclear Factor Kappa B (NF-κB) pathway, has also been linked to the secretion of VEGF and IL-8 in other cancers such as breast [19] and prostate cancer [20]. There is also evidence to suggest that resveratrol may attenuate NF-κB [24]. Therefore, we hypothesised that NF-κB signalling is a key signalling pathway in ovarian cancer and is a potential primary target of resveratrol. In order to investigate the effects of resveratrol and acetyl-resveratrol on NF-κB protein and its phosphorylation (pNF-κB), we employed western blotting and immunofluorescence. We stained frozen sections of SKOV-3 aggregates and OVCAR-5 clusters for the heterodimer NF-κB and pNF-κB. There was an obvious difference in the abundance of NF-κB and pNF-κB between the two cell lines, with SKOV-3 expressing a greater amount of the proteins (Fig. 5). The NF-κB molecule was observed inside the nucleus (red dots) and cytoplasm of SKOV-3 cells (Fig. 5a). The immunostaining of pNF-κB control cells was also located inside the nucleus and cytoplasm with a diffused staining pattern (Fig. 5b). After treatment with resveratrol (Fig. 5c and d) and acetyl-resveratrol (Fig. 5e and f) the abundance of both proteins at the nucleus was reduced. There was a low abundance of NF-κB and pNF-κB in OVCAR-5 cell clusters (Fig. 5g and h).

**Fig. 6:**
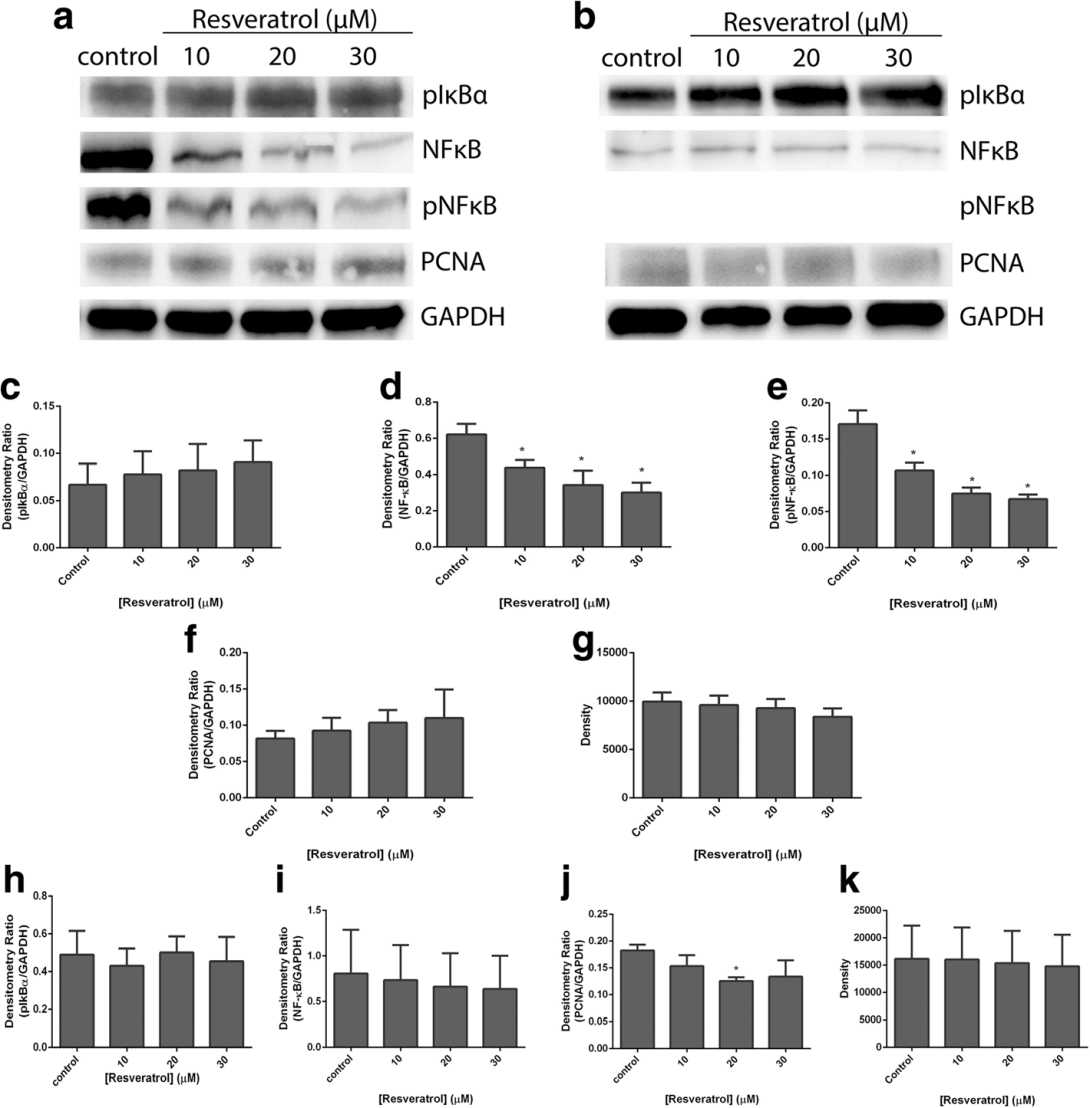
Western blot analysis of SKOV-3 aggregates (**a**) and OVCAR-5 clusters (**b**) treated with resveratrol for 6 days. Densitometry ratios relative to GAPDH of pNF-κB, NF-κB, pIκBα and PCNA of SKOV-3 aggregates (**c**, **d**, **e** and **f**) and of OVCAR-5 clusters (**g**, **h**, **i** and **j**). * indicates statistical significance *p* < 0.05 compared to the control. *n* = 4 independent experiments in triplicate. GAPDH expression is a house keeping protein (**k**)

**Fig. 7:**
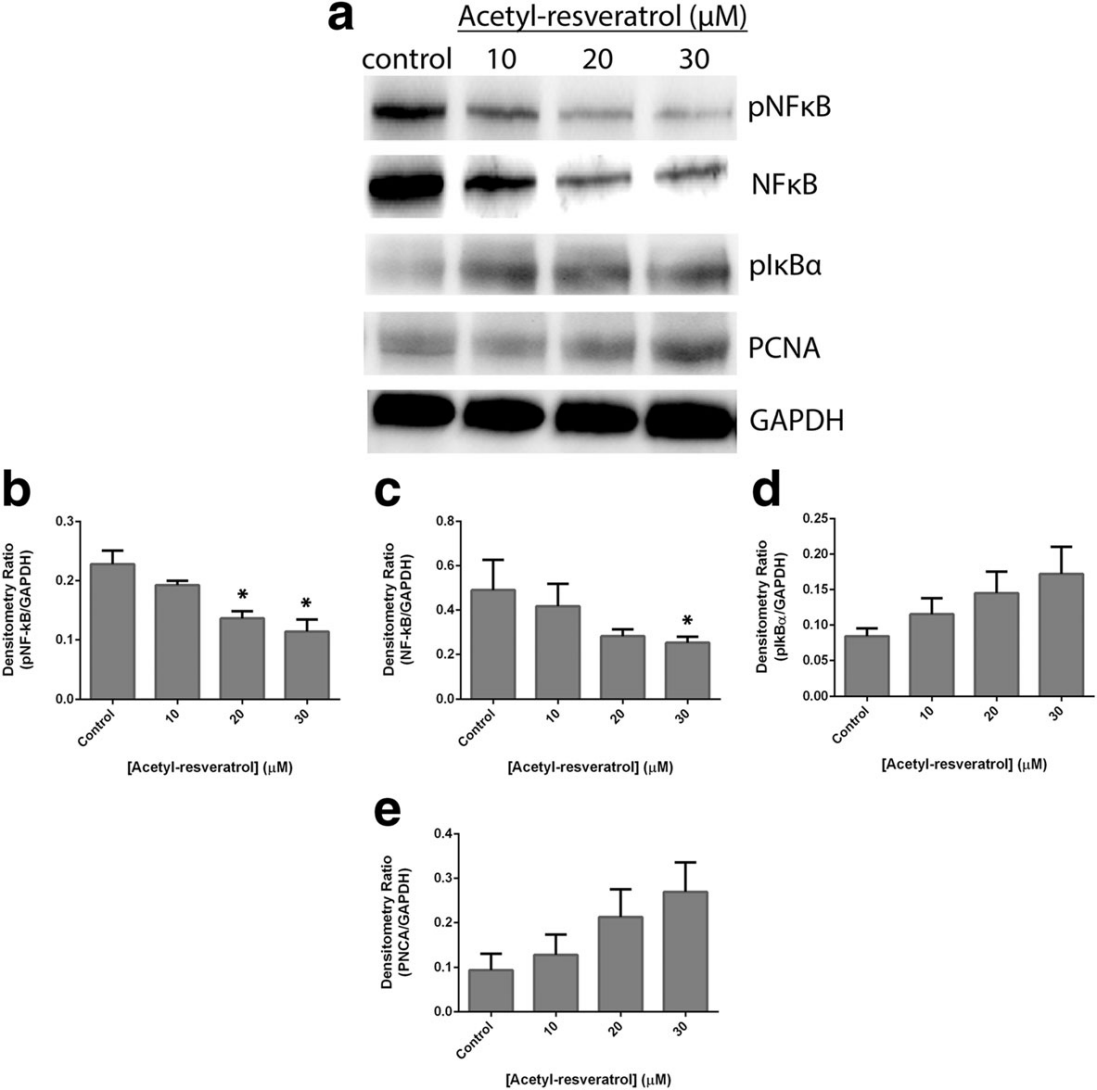
Western blot analysis of SKOV-3 aggregates (**a**) treated with acetyl-resveratrol for 6 days. Densitometry ratios relative to GAPDH of pNF-κB (**b**), NF-κB (**c**), pIκBα (**d**) and PCNA (**e**). * indicates statistical significance *p* < 0.05 compared to the control. *n* = 4 independent experiments in triplicate

To support the immunofluorescent imaging, we then examined the expression and activation of NF-κB after 6 days of treatment with the compounds using western blots. We also investigated two additional proteins that were known to associate with NF-κB; pIκBα and PCNA. As shown in Fig. 6, SKOV-3 cell lysates pNF-κB (Fig. 6a and c) and NF-κB (Fig. 6a and d) showed high expression which were significantly reduced by resveratrol. In OVCAR-5 cell lysates, NF-κB expression was much weaker and unaffected by resveratrol (Fig. 6b and g).and pNF-κB was not detected (Fig. 6b). pIκBα expression (Fig. 6b and h) was in contrast much stronger but was also unaffected by resveratrol. Acetyl-resveratrol at 20 and 30 μM significantly reduced pNF-κB (Fig. 7a and b) and NF-κB (Fig. 7a and c). pIκBα and PCNA were unaffected by the presence of resveratrol or acetyl-resveratrol.

### Attenuation of cell growth and VEGF secretion by a NF-κB inhibitor

**Fig. 8:**
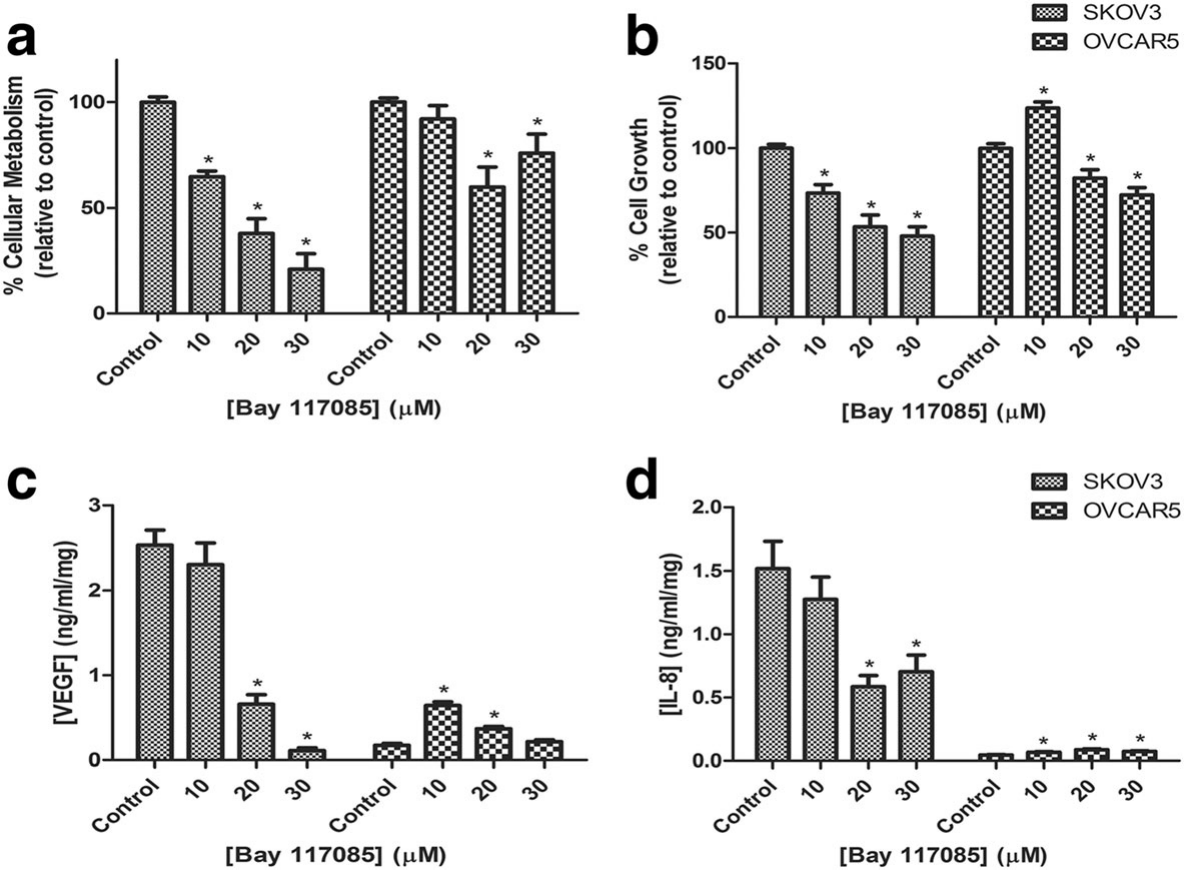
Treatment of SKOV-3 aggregates and OVCAR-5 clusters with Bay 11-7085. Cellular metabolism (**a**) and cell growth (**b**) are relative to control after 6 days treatment. The secretion of VEGF (**c**) and IL-8 (**d**) measured after 6 days of treatment. * indicates statistical significance *p* < 0.05 compared to the control. *n* = 4 independent experiments in triplicate

To assess if the NF-κB protein is associated with cell growth, we employed Bay 11-7085, a specific NF-κB inhibitor. SKOV3 and OVCAR5 aggregates were treated for 6 days with inhibitor at the same concentrations as resveratrol in the previous experiments. Both metabolism and growth of the SKOV3 aggregates was reduced in a dose dependent fashion by as much as 79 and 52%, respectively (Fig. 8a and b). The metabolism and growth of OVCAR5 clusters was also adversely affected, however, the inhibitor was not as effective. Metabolism decreased by only 40% at the most and growth by 28% (Fig. 8a and b). The secretion of VEGF and IL-8 by SKOV3 aggregates was also dose-dependently reduced by the NF-κB inhibitor, 30 μM of the inhibitor decreased VEGF secretion by 96% and IL-8 by 54% when compared to the control (Fig. 8c and d). Conversely, OVCAR5 clusters secreted slightly more VEGF and IL-8 when challenged (Fig. 8c and d).

## Discussion

Of all the gynaecological cancers ovarian cancer is the deadliest. It is very difficult to detect, therefore, three quarters of patients upon diagnosis already have an advanced stage of the disease. The current treatment options for this cancer usually become ineffective after a while and overall survival rates are not good. Finding alternative treatments and targets related to chemoresistance is of the utmost importance.

Advanced ovarian cancer disseminates via cells in ascitic fluid, in which the cells survive by aggregating together [5]. These aggregates then settle at a secondary location and grow into tumours. The morphology of metastasising ovarian cancer cells has been implicated in the development of chemoresistance [6]. Targeting aggregates that are formed by these cells, therefore, could be very useful in the treatment of ovarian cancer. In this study, we grew 3D aggregates of the SKOV-3 and OVCAR-5 cell lines and demonstrate that resveratrol and acetyl-resveratrol are capable of restricting the growth and metabolism of the SKOV-3 aggregates in a time- and dose-dependent manner. We used a 10 μM dose as this concentration can be found in food products and is pharmacologically relevant [29]. Previously we have tested 5, 10, 50 and 100 μM resveratrol and acetyl resveratrol [32] and found that 50 and 100 μM of both compounds was able to reduce cell growth and increase cleaved PARP protein. Therefore, we chose to investigate 20 μM and 30 μM as they are in between the pharmacologically relevant dose and the previously established effective dose. OVCAR-5 cell clusters showed limited responses to resveratrol and its derivative.

Resveratrol has been shown to be capable of reducing cancer growth in previous studies [38–41]. However, these studies use very high doses of the compound, for example Dann et al [38] used 100 μM and Ji et al [39] used up to 200 μM. Furthermore, we have measured the effect of repeat doses over a long time period as we hypothesised that because of the morphology of the ovarian cancer aggregates a much longer exposure time would be required to observe any changes. In contrast, the majority of the studies done to date have been in monolayer cultures using high doses of resveratrol and measuring its effects over short time periods [42–44]. The concentrations of resveratrol and its derivative in our study did not decrease the total expression of PCNA. It may be possible that due to the cell aggregate morphology there are different metabolic cell populations within the aggregates; cells that are at the rim are fully exposed to nutrients and would divide faster than cells inside the aggregates. When these aggregates are exposed to resveratrol and acetyl-resveratrol, cell integrity at the rim may be compromised and that may allow nutrients to penetrate deeper inside the aggregate. As a consequence, this will trigger cells inside the aggregates to express PCNA [45]. Therefore, although there are less cells overall in treated aggregates, PCNA may be expressed by cells further inside the smaller aggregates compared to the large control aggregates, thus, PCNA appears to be unchanged after treatment.

The pathogenesis of ovarian cancer is dependent on various molecules present in peritoneal fluid. VEGF is a key regulator of angiogenesis, a physiological process that is very important to cancer survival, which is associated with cell proliferation [7] and migration [46]. VEGF is highly expressed by ovarian cancer and bystander cells and its concentration is associated with tumour aggression and a poor prognosis [34], as such it is implicated in ovarian cancer pathology. The current study measured the expression of VEGF after treatments with resveratrol and acetyl-resveratrol and found that the VEGF production from SKOV-3 cell aggregates was attenuated by both compounds. The decrease in VEGF secretion correlates with the time- and dose-dependent growth restriction of SKOV-3, this result is in accordance with previous monolayer studies [47–49]. The level of VEGF secretion by OVCAR-5 was, however, very low (on average 8-fold less than SKOV-3) and unaffected by the treatments. This difference between cell lines, although not exclusively studied before, was not totally unexpected. The cell lines were chosen as they have different receptors for adhesion [50] and have different morphology when grown as a monolayer or as 3D cell aggregates or clusters. SKOV-3 cells form large dense aggregates, whereas OVCAR-5 cells form small clusters. The distinct morphology of the cell lines in the 3D model may contribute to the activation of signalling molecules that associate with the transcriptional profile of VEGF.

Unexpectedly the secretion response of IL-8 is the reverse of VEGF. IL-8 is a cytokine that under normal conditions is involved in the inflammatory process, where it attracts and activates neutrophils at the site of infection [35], and is also a potent promoter of angiogenesis [51]. Both of these processes are keys to cancer cell survival and metastasis. Furthermore, IL-8 has been shown to have these effects in ovarian cancer [36, 52]. Huang et al. [53] indicated that VEGF and IL-8 secretion are closely correlated and even potentially controlled by the same intracellular signalling pathway. Additional studies even suggest that IL-8 controls the expression of VEGF [54]. However, the effect we elicited on IL-8 with resveratrol and acetyl-resveratrol is contrary to these suppositions. The increased secretion of IL-8 in response to resveratrol has been observed before in normal human keratinocytes [55]. Potapovich et al. [55] first stimulated the cells with TNFα and then challenged them with 50 μM resveratrol and found that IL-8 secretion was upregulated. Considering all this evidence, we propose that resveratrol is suppressing one signalling pathway that controls VEGF expression whilst possibly up-regulating one that controls IL-8 expression. It is possible that the production of IL-8 in our study conditions may reflect a compensatory mechanism that the cancer cells are using to overcome the lack of VEGF.

In an effort to elucidate the molecules or mechanism targeted by resveratrol and acetyl-resveratrol, we measured the expression of proteins associated with the NF-κB signalling pathway. NF-κB is a family of 5 transcription factors in mammalian cells, RelA (p65), RelB, c-Rel, p50/p105 (NF-κB1) and p52/p100 (NF-κB2), which are able to form homo- and hetero-dimers [15]. The NF-κB cascade involves this family as well as the upstream protein IκBα and toll-like receptors. Phosphorylation of NF-κB is implicated to be important to the transcription of IL-8 [56]. The NF-κB family and signalling pathway promotes proliferation via regulation of genes such as cyclin D1, D3, has been linked to gastric [18], prostate [20] and breast malignancies [19] and has been studied extensively in lymphomas [57–59]. Our data shows that the NF-κB protein is attenuated by resveratrol and acetyl-resveratrol, and the expression of NF-κB observed in the nucleus in the SKOV-3 cell aggregates is markedly reduced. The phosphorylated form of the upstream protein IκBα was unaffected. We have also shown via NF-κB inhibition that the NF-κB protein is involved in the cell growth and secretion of VEGF of the cell lines. The inhibition of NF-κB was twice as effective as resveratrol in reducing cell metabolism, growth and VEGF secretion of the SKOV-3 aggregates suggesting that resveratrol may not be a single action compound, this is further supported by the opposing trends of IL-8 secretion between resveratrol and the NF-κB inhibitor. In OVCAR-5 cell clusters, NF-κB expression is very low and rarely visualized in the nucleus, whilst pNF-κB was below detectable levels, the cell growth was reduced by NF-κB inhibition, however, to a much lesser extent than the SKOV-3 aggregates. Taken together, these results may suggest that resveratrol and acetyl-resveratrol may attenuate the secretion of VEGF via the reduction of NF-κB protein level. It is possible that resveratrol and acetyl-resveratrol may be affecting the NF-κB protein at the transcriptional level. The present study is limited given the lack of growth effects of resveratrol and its derivative in normal ovarian epithelial cells.

## Conclusion

Taken together, our results suggest that resveratrol and acetyl-resveratrol may exhibit anti-ovarian cancer properties through the inhibition of NF-κB. The polyphenols were able to reduce the growth of 3D ovarian cancer cells in a cell line-, concentration- and time-dependant manner. This growth restriction correlated with reduction and reduced nuclear localisation of the NF-κB protein. The reduction of NF-κB in turn correlated with the attenuation of VEGF secretion. Further studies of the anti-ovarian cancer growth activity of resveratrol and acetyl-resveratrol are warranted especially using in vivo animal models before clinical trials.
